# Translational Insights into Extremely Low Frequency Pulsed Electromagnetic Fields (ELF-PEMFs) for Bone Regeneration after Trauma and Orthopedic Surgery

**DOI:** 10.3390/jcm8122028

**Published:** 2019-11-20

**Authors:** Sabrina Ehnert, Steffen Schröter, Romina H. Aspera-Werz, Wiebke Eisler, Karsten Falldorf, Michael Ronniger, Andreas K. Nussler

**Affiliations:** 1 Siegfried Weller Institute for Trauma Research, Depterment of Trauma and Reconstructive Surgery, BG Unfallklinik Tübingen, Eberhard Karls Universität Tübingen, D-72076 Tübingen, Germany; sschroeter@bgu-tuebingen.de (S.S.); rominaaspera@hotmail.com (R.H.A.-W.); weisler@bgu-tuebingen.de (W.E.); andreas.nuessler@gmail.com (A.K.N.); 2 Sachtleben GmbH, Hamburg, Haus Spectrum am UKE, Martinistraße 64, D-20251 Hamburg, Germany; falldorf@citresearch.de (K.F.); ronniger@citresearch.de (M.R.)

**Keywords:** extremely low frequency pulsed electromagnetic fields (ELF-PEMF), bone regeneration, fracture healing, spinal fusion, osteoporosis, bone mineral density, osteoarthritis, pain, bone cells

## Abstract

The finding that alterations in electrical potential play an important role in the mechanical stimulation of the bone provoked hype that noninvasive extremely low frequency pulsed electromagnetic fields (ELF-PEMF) can be used to support healing of bone and osteochondral defects. This resulted in the development of many ELF-PEMF devices for clinical use. Due to the resulting diversity of the ELF-PEMF characteristics regarding treatment regimen, and reported results, exposure to ELF-PEMFs is generally not among the guidelines to treat bone and osteochondral defects. Notwithstanding, here we show that there is strong evidence for ELF-PEMF treatment. We give a short, confined overview of in vitro studies investigating effects of ELF-PEMF treatment on bone cells, highlighting likely mechanisms. Subsequently, we summarize prospective and blinded studies, investigating the effect of ELF-PEMF treatment on acute bone fractures and bone fracture non-unions, osteotomies, spinal fusion, osteoporosis, and osteoarthritis. Although these studies favor the use of ELF-PEMF treatment, they likewise demonstrate the need for more defined and better controlled/monitored treatment modalities. However, to establish indication-oriented treatment regimen, profound knowledge of the underlying mechanisms in the sense of cellular pathways/events triggered is required, highlighting the need for more systematic studies to unravel optimal treatment conditions.

## 1. Background

It is well accepted that bone is a mechanosensory organ, which requires continuous strain to preserve its functional structure and prevent disuse bone loss (osteopenia or osteoporosis). The resulting premise that bones constantly adapt to meet their mechanical demands is referred to as Wolff’s law [1]. In the 1960s, it was first reported that mechanical strain alters the electrical potential along the lateral and longitudinal axes of compact bone, providing local stimuli for bone-forming cells [2]. Bassett and colleagues suggest collagen piezoelectricity as a potential underlying mechanism. According to their theory, applied stress generates local potential gradients along the collagen fibers [3,4]. This mechanism, ascribed to the non-centrosymmetric nature of collagen, is well accepted for dry bone tissue [5]. However, for wet bone tissue, there was more and more experimental evidence that fluid-induced shear stress and associated streaming potentials cause the strain-generated potentials [6]. Offering a possible explanation of how bone is selectively deposited at mechanically challenged areas, these described phenomena raised hope in the scientific community that these mechanisms can be utilized to support bone function and fracture healing. Anecdotal reports that electromagnetic fields fostered healing of persistent non-union fractures further fueled the interest in this area [7,8,9,10]. In these studies, mainly extremely low frequency pulsed electromagnetic fields (ELF-PEMFs) have been applied.

**Figure 1:**
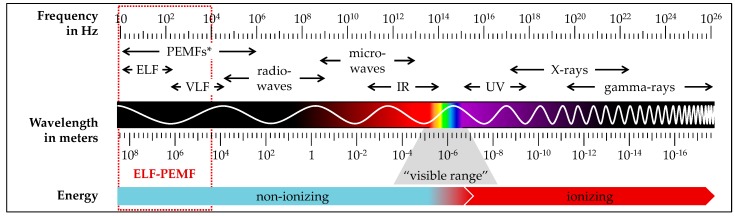
The electromagnetic spectrum with its characteristic frequencies, wavelengths, and electromagnetic energy. The electromagnetic spectrum ranges from extremely long wavelengths with small frequencies (e.g., ELFs (extremely low frequency) and VLFs (very low frequency)) to extremely short wavelengths with high frequencies (e.g., UV (ultraviolet), x-rays or gamma-rays). Depending on the wavelength and frequency, it comprises both nonionizing and ionizing radiation. * In case for pulsed electromagnetic fields (PEMFs), “frequency” and “repetition rate” are often used synonymously, knowing that lower-frequency electromagnetic pulses are super-positioned by higher frequencies, yielding a wider frequency spectrum. The range of extremely low frequency pulsed electromagnetic fields (ELF-PEMFs) considered in this review is marked in red.

ELF-PEMFs represent a subclass of electromagnetic fields. With frequencies or repetition rates (often used synonymously when describing PEMFs) up to a few hundred Hz, ELF-PEMFs are situated at the lower end of the electromagnetic spectrum (Figure 1).

**Figure 2:**
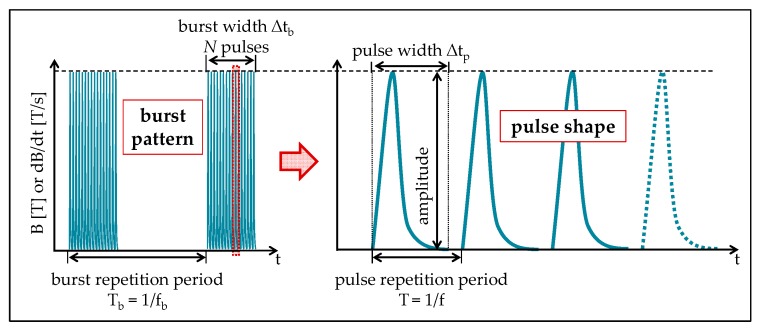
Schematic overview of the terms used for characterizing bursts and pulses of ELF-PEMF. PEMF-signals use periodically repeated bursts consisting of a certain number of pulses, N, at a certain frequency, f. The pulses can be described by their shape, amplitude, and pulse width, $\Delta t_{p}$. Here, bursts are repeated at the burst-frequency $f_{b}\ll f$. Some publications refer to the magnetic flux density, B, or the time-derivative of the magnetic field, dB/dt, others only state the search coil induction voltage. However, without knowing the exact search coil dimensions, the magnetic field amplitude cannot be derived from this value.

However, the Fourier-frequency spectrum of a signal can range from extremely low (≤300 Hz) to high frequencies (1 kHz to ≤1MHz), whereas the latter reflects the higher frequency pulse repetition rate *f* (for definition, see Figure 2). In pulsed EMF (PEMF), bursts of pulses are sent in on–off periods. The extremely low frequency (ELF) notation can reflect the burst or the pulse repetition rate. ELF-PEMF radiation is nonionizing and uses electrical energy to direct a series of magnetic pulses through biological tissue. Each of the magnetic pulses induces a tiny electrical signal in the exposed tissue that is thought to stimulate tissue repair without inducing significant thermal effects [11].

## 2. In Vitro Evidence for ELF-PEMF Effects on Bone Cells

Within the bioelectromagnetic science society, certain theories on how natural and artificial ELF-PEMF may induce cellular effects on the molecular level are discussed, for example, the molecular gyroscope model [12], Lorentz models [13,14], DNA antenna model [15], radical pair model [16], and ion cyclotron resonance [17]. Cells in the human body are continuously exposed to electrical charges (e.g., Na^2+^, K^+^, or Cl^−^ ion gradients, which regulate cellular membrane potentials) involved in a manifold of cellular processes [18]. Therefore, it is also feasible that ELF-PEMFs influence cellular responses by influencing these natural ion gradients, either passively by ionic forces or actively by regulating so-called voltage-gated ion channels [19,20,21]. However, it might well be that the effects triggered by ELF-PEMFs can be only explained by a combination of these theories. Focusing on the bone, studies have demonstrated that ELF-PEMF treatment is reported to cause calcium flux, induce RNA expression, stimulate synthesis of extracellular matrix proteins and growth factors, and initiate signaling cascades involved in viability, proliferation, and differentiation. Some of these ELF-PEMF effects on viability, growth, and function of bone cells will be described in more detail in the following paragraphs. 

### 2.1. ELF-PEMF Effects on Viability of Bone Cells

Since 50 years ago the first suspicion arose that electromagnetic fields, especially those created by 50/60 Hz power lines, may cause possible health risks [22], many in vitro experiments frequently addressed the question, whether ELF-PEMFs affect cell viability. Considering that ELF-PEMFs are located at the lower, nonionizing, and nonthermal range of the electromagnetic spectrum, a direct temperature-associated damage of DNA or proteins may be excluded. Several studies have shown that ELF-PEMF treatment may induce formation of reactive oxygen species (ROS) [23,24], which may affect cell viability. Accumulation of ROS or oxidative stress, causing upregulation of heat shock proteins and direct damage of the DNA, was mainly observed when cells are exposed to EMFs in the micro- and radio-frequency range [25]. However, Chang et al. showed ROS induction in osteoclasts exposed to ELF-PEMF, which significantly enhanced apoptosis in these cells, especially with prolonged treatment durations [26]. Contrarily, Tang and Zhao showed reduced apoptosis rates in primary mouse osteoblasts and ROS cells exposed to ELF-PEMF (f = 50 Hz) [27]. This might be partly explained by our own study, which showed ROS (mainly •O_2_^−^and H_2_O_2_) formation caused by repetitive ELF-PEMF exposure (f = 16 Hz) induced expression and activity of antioxidative enzymes, for example, superoxide dismutase (SOD), catalase (CAT), glutathione peroxidase (GPX), and glutathione-disulfide reductase (GSR). These enzymes, being involved in the mitochondrial degradation of ROS, are essential for the survival of organisms and their health [28]. In line with this, the work of Raggi et al. showed that repetitive exposure (27 min per day for 10 days) to ELF-PEMF reduced oxidative stress measures in blood of healthy volunteers for up to 1 month after the treatment [29]. Considering that ROS are generally produced as by-products of the mitochondrial respiratory chain, increased ROS levels after ELF-PEMF exposure might indicate towards increased proliferation or enhanced function in these cells.

### 2.2. ELF-PEMF Effects on Bone Cell Growth

Zhang et al. investigated the effect of f = 15 Hz EMFs with different waveforms, namely rectangular (ELF-REMF), triangular (ELF-TEMF), sinusoidal (ELF-SEMF), and ELF-PEMF, on proliferation of primary rat calvaria cells. In their experiments, only ELF-PEMF and ELF-REMF induced proliferation. ELF-SEMF even harmed cell growth [30]. This is in line with the work of Zhou et al., who showed comparable effects in primary rat calvaria cells exposed to f = 50 Hz ELF-SEMF, ELF-TEMF, and ELF-REMF [31]. This, in turn, suggests that altering the frequency (f = 15 vs. 50 Hz) less strongly affects bone cell proliferation than altering the waveform. This assumption is supported by our work, where ELF-PEMFs with different frequencies in the range from f = 10 to 90.6 Hz all induced proliferation (~50%) of primary human osteoblasts in equal measures [32]. The work of Tang and Zhao showed an increase in S phase in primary mouse osteoblasts and ROS cells after exposure to f = 50 Hz ELF-PEMF [27], indicating towards an increase in cell proliferation. In line with this, Wei et al. showed that exposure to f = 48 Hz ELF-PEMF promoted proliferation (increased number of cells in S and G(2)M phase) of primary rat calvaria cells but not of MC3T3-E1 cells [33], suggesting that the observed effect is strongly dependent on the differentiation status of the cells. Bique et al. observed similar effects in strongly differentiated SaOs-2 cells and less differentiated MC3T3-E1 cells exposed to f = 50 Hz ELF-PEMF [34]. This finding is supported by the work of Kaivosoja et al., which showed stimulatory effects of f = 15 Hz ELF-PEMF on proliferation being more pronounced in SaOs-2 cells when compared with less differentiated mesenchymal stem cells [35]. Yamaguchi et al. proposed that ELF-PEMF-dependent alterations in intercellular gap junction communication might be responsible for this effect, as in their experiments, exposure to a f = 120 Hz ELF-PEMF rapidly (within 1 h) decreased intercellular gap junction communication only in immature MC3T3 cells, but not in more mature cell types. Interestingly, this phenomenon was not dependent on the applied frequencies (f = 30, 60, or 120 Hz) but linearly correlated with the intensity of the fields [36]. This finding is supported by the work of Lohmann et al., which showed changes in Connexin 43 levels in ROS 17/2.8 and MLO-Y4 cells within 3 days following f = 15 Hz ELF-PEMF exposure [37].

### 2.3. ELF-PEMF Effects on Bone Cell Function

Zhou et al. not only showed a waveform-dependent effect of ELF-PEMFs (f = 50 Hz) on proliferation, but also on osteogenic differentiation of primary rat calvaria cells. In their experiments, osteoblastic markers, e.g., alkaline phosphatase (ALP) and mineralized matrix, were induced only by ELF-TEMF and to a lesser extent by ELF-SEMF [31]. Comparable results were observed by Zhang et al., who showed enhanced matrix mineralization only in primary rat calvaria cells exposed to f = 15 Hz ELF-TEMF and ELF-PEMF, but not to ELF-SEMF [30]. The authors suggest extracellular calcium, P2 receptor on the membrane, and phospholipase C pathway being involved in the observed effects of the ELF-PEMF treatment [30]. ELF-PEMF modulation of calcium influx is reported by several studies, proposedly via voltage-gated calcium channels [38,39,40,41,42]. Increased calcium influx, in turn, may activate specific potassium channels [19]. Comparable ELF-PEMF effects have been reported for a large variety of ion channels and membrane receptors involved in membrane trafficking (for overview, see [43]). Resulting alterations in ion gradients affect several intracellular phenomena, for example, cell volume or signal transduction [19]. For example, Sollazzo et al. showed activation of protein kinase B and signal transducer and activator of transcription (STAT) 3 (both known to regulate bone metabolism [44,45,46]) signaling in MG-63 cells exposed to f = 75 Hz ELF-PEMF [47]. Furthermore, ELF-PEMF exposure not only induced expression of transforming growth factor beta (TGF-β) and bone morphogenetic proteins (BMPs), but also enhanced their signaling [48,49,50,51,52,53]. Similar was observed for the Wnt/β-catenin signaling pathway [40,54,55,56,57,58], known to be strongly activated by calcium influx. However, Wnt, TGF-β, and BMP signaling may also be regulated by the cells’ primary cilia [59,60], which are cellular structures reported to be affected by ELF-PEMF [43,48,61,62]. Therefore, it is feasible that ELF-PEMF affect primary cilia structure and function.

In our study, increased osteogenic function (ALP activity and matrix mineralization) was associated with an activation of extracellular signal–regulated kinase (ERK) 1/2 signaling [32], proposedly induced by ROS [24]. In certain bone modeling phases, ROS may activate osteoclasts while inhibiting osteoblasts [63]. By inducing ROS, ELF-PEMF thus may favor osteoclastogenesis, an assumption supported by the studies of Pi and Zhang [64,65]. However, there are also several other reports showing ELF-PEMF inhibitory effects on osteoclastogenesis [66,67,68]. He et al. suggest that ELF-PEMF suppressive effects are mediated by endocrine effects of osteoblasts [67], a hypothesis supported by several other studies [61,69,70,71,72]. While Wang et al. proposed that regulation of osteoclastogenesis is strongly dependent on the intensity of the applied ELF-PEMF [73], Lei et al. showed bone metabolic ELF-PEMF effects in osteoporotic (ovariectomized) mice which strongly depended on the frequency range applied [74]. While lower frequencies induced osteoblast function, higher frequencies inhibited osteoclast function [74]. This is in line with our previous work, showing that the ELF-PEMF with a frequency of f = 16 Hz, which most effectively induced osteoblast function, did not affect osteoclast function [32]. Applying the same ELF-PEMF with an only 10 Hz higher frequency, however, resulted in an increased osteoclast function [75].

Bagheri et al. showed that continuous exposure to f = 75 Hz ELF-PEMF increased the expression of ALP, Runx2, and Osterix [76]. In line with this, Sollazzo et al. showed increased expression of fibronectin (FN), vinculin (VCL), collagen (COL1A2), osteonectin, and tissue inhibitor for matrix-metalloproteinase 1 (TIMP1) in MG-63 cells exposed to f = 75 Hz ELF-PEMF [47]. At the same time, expression of proteins involved in extracellular matrix (ECM) degradation (e.g., matrix-metalloproteinase 11 (MMP11)) decreased in these cells [47]. These results might be partly explained by the work of Blank and Goodman, which identified specific EMF-responsive DNA sequences, so-called nCTCTn sequences, which are thought to regulate expression of genes (e.g., c-myc or hsp70) as an immediate and direct response to ELF-PEMF exposure [77,78].

Increased expression of osteogenic marker genes was accompanied with increased osteoblast function. The work of Lu et al. showed stimulatory effects of f = 20 Hz ELF-PEMF on ALP and Osteocalcin levels in rat-derived mesenchymal stem cells. In their experiments, ELF-PEMF exposure even suppressed adipogenic differentiation [79]. In line with this, both the study of Martino et al. [80] and the study of Hannay et al. [81] showed f = 15 Hz ELF-PEMF stimulatory effects on ALP activity (within few hours) and matrix mineralization (within 2–4 days) of SaOs-2 cells. These results were comparable to our study, which showed a frequency-dependent increase in ALP activity and matrix mineralization in ELF-PEMF-exposed primary human osteoblasts [32].

Interestingly, in our study, the observed ELF-PEMF effect was most pronounced in cells with poor basal osteogenic function, suggesting that ELF-PEMF treatment might be most efficient in conditions where bone formation is somehow suppressed [32]. However, this assumption requires further investigation. Examples are the studies of Cai et al. [82], Li et al. [83], or Jing et al. [84], which investigated the effect of ELF-PEMF on architecture and mechanical properties of bone in diabetic animals, known to have skeletal deficiencies, or the studies of Lei et al. [74,85], Li et al. [86], Zhou et al. [87,88], or Androjna et al. [89], which investigated the effect of ELF-PEMF on bone quality in osteoporotic animals.

## 3. Clinical Studies on the Effect of ELF-PEMF Treatment on Bone

In vitro studies and initial case reports hyped the use of ELF-PEMFs to support fracture healing and bone function starting in the 1970s, resulting in continuously increasing numbers of publications on the proposed subject (for overview, see search strategy at the end of the manuscript). However, a closer look on the studies available regarding ELF-PEMFs’ bone effects quickly attenuates the hype. As claimed by existing systematic reviews and meta-analyses, many of these reports lack placebo controls [90,91,92,93,94,95,96,97,98]. Therefore, these studies can be counted as tolerance studies at best. However, a sizeable amount of prospective studies with adequate controls remains that can be used to judge possible effects of ELF-PEMFs on bone. These studies are summarized in the following paragraphs.

### 3.1. ELF-PEMF Treatment for Pseudarthrosis and Non-Union Fractures

**Figure 3:**
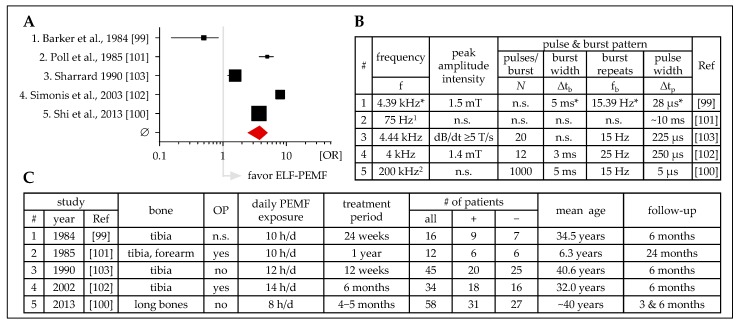
Overview on clinical studies (prospective, placebo-controlled, blinded) investigating the effects of ELF-PEMF treatment on healing of pseudarthrosis/non-union fractures. (**A**) Forest plot showing OR ± 95% C.I.—sum was calculated as described by Neyeloff et al. [107], with weighting based on sample size. (**B**) Tabular overview of the ELF-PEMF used in the presented studies. (**C**) Tabular overview of the presented studies including affected bones, number of patients, treatment, and follow up. ^1^ BIOSTIM^®^, IGEA S.p.A., Carpi, Italy; ^2^ Orthopulse^®^ II, OSSATEC, Uden, Netherlands; f: frequency; $\Delta t_{b}$: burst width; $f_{b}$: burst repeats; $\Delta t_{p}$: pulse width; OP: surgery; + ELF-PEMF treatment; − placebo treatment; n.s.: not specified; * obtained from secondary source.

There are only five studies on pseudarthrosis and fracture non-unions which compare ELF-PEMF treatment with placebo treatment in a prospective and blinded fashion (Figure 3). These studies use ELF-PEMFs with frequencies ranging from 15 Hz [99] to 200 kHz [100]. Two studies used ELF-PEMF generators approved for medical use—BIOSTIM® (IGEA S.p.A., Carpi, Italy) [101] and Orthopulse® II (OSSATEC, Uden, Netherlands) [100]. ELF-PEMF exposures were between 8 and 14 hours per day for 3 to 12 months. Despite the different treatment conditions, ELF-PEMF treatment overall was able to induce healing of pseudarthrosis and non-union fractures (mean OR = 3.70 ± 1.02). At first glance, it may seem that the higher frequencies applied were more effective than the lower frequencies applied. However, this cannot be generalized as ELF-PEMF parameters (e.g., field intensities) vary. Furthermore, other factors, for example, the patients’ age or the persistence of the bone defect (≥1 year vs. 4–6 months) have to be considered too. Overall, the most consistent results were obtained when ELF-PEMF treatment was started early during the development of a pseudarthrosis (delayed-union) [100]. The most pronounced effects, however, were observed when ELF-PEMF treatment was applied to patients with a pseudarthrosis persisting for ≥1 year [102]. Although four of the studies were comparable for patients’ age and follow-up time-points, the diversity in ELF-PEMF characteristics applied prevents reliable predictions on optimal treatment duration [99,100,102,103]. This is in contrast to the follow-up study, which summarized clinical outcomes of 1382 patients who received ELF-PEMF treatment (∆t_b_ = 4.5 ms; f_b_ = 15 Hz; peak amplitude intensity = 1.6 mT; ∆t_p_ = 225 μs; generated with the EBI Bone Healing System; Zimmer Biomet, Warsaw, IN, USA) to support healing of acute, delayed, and non-union fractures. In this study, fracture healing was gradually accelerated with elongation of the daily exposure time, for example, fracture healing was significantly faster when the field was applied for ≥9 h/d when compared with <3 h/d [104]. In contrast to the above-mentioned five prospective studies, this retrospective study refers to a larger study cohort. While the five prospective studies attempted to match the placebo-treated and the ELF-PEMF-treated patients regarding possible confounding factors, for example, age, nutritional status, alcohol or cigarette consumption, comorbidities (e.g., diabetes mellitus), or medication (e.g., corticosteroids or nonsteroidal anti-inflammatory drugs) [105,106], this retrospective study lacks information on these patient characteristics, therefore, the conclusion that elongated ELF-PEMF exposure times are favorable for bone healing has to be handled with care. It might well be that therapy compliance decreased with an increasing number of confounding factors. et al.

### 3.2. ELF-PEMF Treatment to Support Acute Fracture Healing

**Figure 4:**
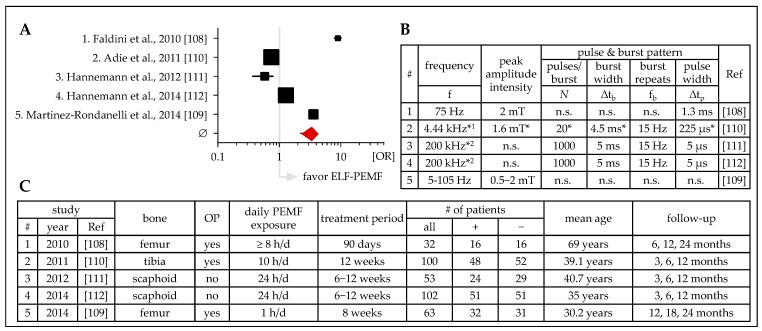
Clinical studies (prospective, placebo-controlled, blinded) investigating the effects of ELF-PEMF treatment on healing of acute fractures. (**A**) Forest plot showing OR ± 95% C.I.—sum was calculated as described by Neyeloff et al. [107], with weighting based on sample size. (**B**) Overview on the ELF-PEMF used in the presented studies. (**C**) Overview on the presented studies including affected bones, number of patients, treatment, and follow up. ^1^ EBI Bone Healing System, Zimmer Biomet, Warsaw, IN, USA; ^2^ Bone Growth Stimulator, OSSATEC, Uden, Netherlands; f: frequency; $\Delta t_{b}$: burst width; $f_{b}$: burst repeats; $\Delta t_{p}$: pulse width; OP: surgery; + ELF-PEMF treatment; − placebo treatment; n.s.: not specified; * obtained from a secondary source.

Similarly, there are five prospective and blinded studies investigating the effect of ELF-PEMF treatment on acute fracture healing for the femur [108,109], tibia [110], or scaphoid [111,112] (Figure 4). These studies use ELF-PEMFs with fixed frequencies [108,110,111,112] or a frequency range (5–105 Hz [109]). Three studies used ELF-PEMF generators approved for medical use—EBI Bone Healing System (Zimmer Biomet, Warsaw, IN, USA) [110] and Bone Growth Stimulator (OSSATEC, Uden, Netherlands) [111,112]. Daily ELF-PEMF exposures were even more diverse than in the studies before and ranged from 1 to 24 hours per day for periods of 42 to 90 days. Four studies were comparable regarding the patients’ mean age (30 to 41 years vs. 69 years). Three studies considered earlier follow-up time-points (3 to 12 months). The other two studies considered later follow-up time-points (6 to 18 months and 12 to 24 months). Summarizing the five studies at the common 12 month follow-up time-point, ELF-PEMF treatment was able to support fracture healing (mean OR = 2.86 ± 0.59). At the later time-points, the studies that applied higher-frequency ELF-PEMFs seemed to be more effective, while at the earlier follow-up time-points, the studies that applied lower-frequency ELF-PEMFs were able to further accelerate fracture healing [110,111,112]. Strongest effects were observed with an ELF-PEMF treatment of 75 Hz applied for ≥8 hours per day for 90 days [108], however, it is not clear whether this effect is attributed to the higher frequency, other specific ELF-PEMF characteristics, and/or the higher age. Interestingly, solid results were observed when applying the ELF-PEMF with a range of frequencies—with this ELF-PEMF, a daily exposure of 1 hour was sufficient to induce fracture healing [109]. In line with this, the study of Cheing et al. showed that 30 minutes ELF-PEMF (∆t_b_ = n.s.; f_b_ = 50 Hz; peak amplitude intensity = 9.9 mT; ∆t_p_ = n.s.; generated with the Pulsed Magnetic Field Therapy System—model XKC-660W; Magnetopulse International, Griffin, Australia) exposure daily for 5 days enhanced the effect of cooling—effectively reducing swelling and pain, this early (even before surgery) ELF-PEMF treatment significantly accelerated healing of radius fractures [113]. Similarly, Lazovic et al. were able to improve functional outcome after radius fracture with only 30 minutes ELF-PEMF (∆t_b_ = n.s.; f_b_ = 25 Hz; peak amplitude intensity = 6 mT; ∆t_p_ = n.s.; generated with the Elec System, Elbtal, Germany) exposure daily for 10 days [114]. Knowing that very long and complicated treatment procedures reduce the patients’ compliance [115], this study challenges the long daily exposure times and treatment duration used with the other studies.

### 3.3. ELF-PEMF Treatment to Support Healing of Osteotomies

**Figure 5:**
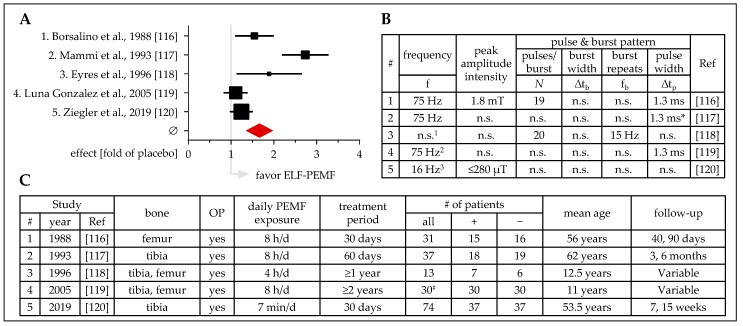
Clinical studies (prospective, placebo-controlled, blinded) investigating the effect of ELF-PEMF treatment on osteotomies. (**A**) Forest plot showing ELF-PEMF effects fold of placebo ± 95% C.I.—sum was calculated as described by Neyeloff et al. [107], with weighting based on sample size. (**B**) Overview on the ELF-PEMFs used in the presented studies. (**C**) Overview on the presented studies including affected bones, number of patients, treatment, and follow up. ^1^ device from Electro-Biology Inc, Fairfield, NJ, USA; ^2^ BIOSTIM^®^, IGEA S.p.A., Carpi, Italy; ^3^ Somagen^®^, Sachtleben GmbH, Hamburg, Germany; f: frequency; $\Delta t_{b}$: burst width; $f_{b}$: burst repeats; $\Delta t_{p}$: pulse width; # left-right comparison; OP: surgery; + ELF-PEMF treatment; − placebo treatment; n.s.: not specified; * obtained from secondary source.

Osteotomies causing a defined gap of the bone deserve a discrete consideration. So far, five studies can be cited which investigated ELF-PEMF treatment to support osteotomy healing, in a prospective and blinded way [116,117,118,119] (Figure 5). Three studies used an ELF-PEMF with a frequency of 75 Hz for 8 hours per day, over a time period of 1, 2, or ≥23 months [116,117,119]. The fourth study used an ELF-PEMF with a frequency of 15 Hz for 4 hours per day for more than 1 year during limb lengthening [118]. The fifth study used an ELF-PEMF with a frequency of 16 Hz for only 7 minutes per day for 30 days following high tibial osteotomy [120]. In three studies, commercial ELF-PEMF devices were used, namely EBI stimulator (Electro-Biology Inc, Fairfield, NJ, USA) [118], BIOSTIM® (IGEA S.p.A., Carpi, Italy) [119], and Somagen® (Sachtleben GmbH, Hamburg, Germany) [120].

These osteotomy-based studies have the advantage that the patients are highly homogeneous and compliant. However, with an expected 100% consolidation rate, readout parameters and follow-up time-points require critical attention. Four of the studies measured consolidation rates and/or the time to heal. Three of the studies additionally investigated the ELF-PEMF effect on bone mineral density (BMD). Overall, ELF-PEMF treatment accelerated osteotomy healing, an effect best observed at earlier follow-up time-points. For example, in the study of Borsalino et al., consolidation was significantly advanced (2.6-fold) in the ELF-PEMF group at day 40, but no further changes were observed at day 90 [116]. This was comparable to the study of Mammi et al., where consolidation rates were significantly advanced in the ELF-PEMF group 60 days following surgery [117]. Extrapolating the results of the study from Ziegler et al., consolidation of the osteotomy gap was accelerated by approx. 5 weeks (~17% reduction). This is in line with the study of Luna Gonzalez et al., where ELF-PEMF exposure reduced the time until removal of the external fixator after limb lengthening by approximately 10% [119]. Furthermore, later follow-up time-points revealed a positive effect of the ELF-PEMF treatment on BMD [116,118,119]. This was also observed by Abdelrahim et al., who found increased BMD after ELF-PEMF treatment 2 hours per day for 12 days (∆t_b_ = 200 ns; f_b_ = 72 Hz; peak amplitude intensity = n.s.; ∆t_p_ = n.s.) in patients with mandibular fractures [121], and Dallari et al., who found increased BMD in patients with hip revision prostheses after ELF-PEMF treatment 6 hours per day for 90 days (∆t_b_ = 5 ms; f_b_ = 75 Hz; peak amplitude intensity = n.s.; ∆t_p_ = 5 µs; generated with the BIOSTIM, IGEA, Carpi, Italy) [122].

### 3.4. ELF-PEMF Treatment for Osteoporosis

**Figure 6:**
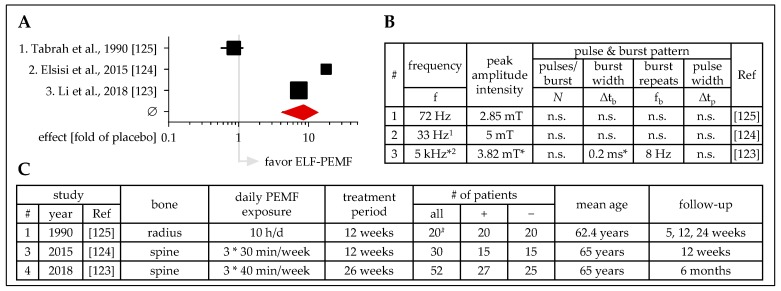
Clinical studies (prospective, placebo-controlled, blinded) investigating the effect of ELF-PEMF treatment on osteoporosis. (**A**) Forest plot showing ELF-PEMF effects fold of placebo ± 95% C.I.—sum was calculated as described by Neyeloff et al. [107], with weighting based on sample size. (**B**) Tabular overview of the ELF-PEMF used in the presented studies. (**C**) Tabular overview of the presented studies including affected bones, basic information on the treatment and follow up, as well as the number of patients investigated. ^1^ Automatic PMT Quattro Pro, ASA magnetic field, Arcugnano, Italy; ^2^ XT-2000B therapeutic stimulator, Tianjin xtmed, Tianjin, China; f: frequency; $\Delta t_{b}$: burst width; $f_{b}$: burst repeats; $\Delta t_{p}$: pulse width; ^#^ left-right comparison; + ELF-PEMF treatment; - placebo treatment; n.s.: not specified; * obtained from a secondary source.

Three studies investigated the use of ELF-PEMF to treat osteoporosis in a prospective and blinded fashion (Figure 6). These studies used ELF-PEMF with frequencies of 8 Hz [123], 33 Hz [124], and 72 Hz [125]. ELF-PEMF treatment conditions reached from 30 minutes per day, 3 days a week for 3 or 6 months up to 10 hours daily for 3 months. Despite the different treatment conditions, ELF-PEMF-treated patients had overall improved BMDs in these studies (mean fold of placebo = 8.62 ± 3.85). Interestingly, the ELF-PEMF effect was more pronounced when using lower frequencies with shorter treatment duration. This finding is supported by the work of Lui et al., which shows that the positive effect of ELF-PEMF treatment, applied 6 times, 40 minutes per week for 5 weeks (∆t_b_ = 0.2 ms; f_b_ = 8 Hz; peak amplitude intensity = 3.82 mT; ∆t_p_ = n.s.; generated with a XT-2000B therapeutic stimulator, Tianjin xtmed, Tianjin, China), on BMD is even comparable to alendronate treatment [126]. In addition to the improved BMD, Li et al. reported that ELF-PEMF treatment additionally reduced marrow fat in these patients [123].

### 3.5. ELF-PEMF Treatment after Spinal Fusion

**Figure 7:**
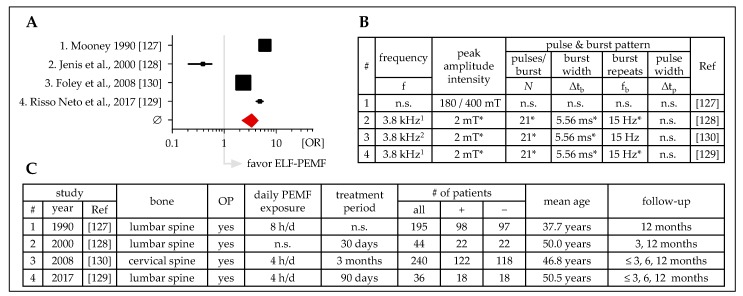
Clinical studies (prospective, placebo-controlled, blinded) investigating the effect of ELF-PEMF treatment on spinal fusion. (**A**) Forest plot showing OR ± 95% C.I.—sum was calculated as described by Neyeloff et al. [107], with weighting based on sample size. (**B**) Tabular overview of the ELF-PEMF used in the presented studies. (**C**) Tabular overview of the presented studies including affected bones, basic information on the treatment and follow up, as well as the number of patients investigated. ^1^ Spinal-Stim, Orthofix Inc., McKinney, TX, USA; ^2^ Cervical-Stim, Orthofix Inc., McKinney, TX, USA; f: frequency; $\Delta t_{b}$: burst width; $f_{b}$: burst repeats; $\Delta t_{p}$: pulse width; OP: surgery; + ELF-PEMF treatment; − placebo treatment; n.s.: not specified; * obtained from a secondary source.

Up to now, there are four prospective and blinded studies investigating the effect of ELF-PEMF treatment on spinal fusion (Figure 7). While one study does not specify the used ELF-PEMF [127], the other studies use ELF-PEMFs with burst frequencies of 15 Hz [128,129,130]. Daily ELF-PEMF exposures ranged from 4 to 8 hours for 30 or 90 days. The patients’ mean age ranged from 37.7 to 50.5 years. In summary, these studies favor the use of ELF-PEMF to support spinal fusion (mean OR = 3.40 ± 1.19). Besides improved spinal fusion, in the study of Omar et al., patients reported pain relief (decrease in VAS (visual analogue scale) score [131]), less disability due to pain (reduced OSW (Oswestry disability index) score [132]), and improved flexion of the lower extremities after ELF-PEMF treatment for 20 minutes per day for 3 weeks (∆t_b_ = n.s.; f_b_ = 4–4000 Hz; peak amplitude intensity = 0.5–1.5 mT; ∆t_p_ = n.s.) [133].

### 3.6. ELF-PEMF Effects on Osteoarthritis

Noteworthy, there are 12 prospective and blinded studies investigating ELF-PEMF treatment as a possible adjunct to conservative treatment of osteoarthritis of the knee (Table 1). By using frequencies all over the ELF-PEMF range, these studies conspicuously differ from the studies reported above. Furthermore, treatment duration, which is quite comparable among these studies (≤1 hour per day for 2 to 6 weeks), is much shorter compared with most studies on bone healing. In contrast to the other studies, which mainly used radiologic endpoints, these studies used different questionnaires for primary outcomes. Pain, for example, was primarily assessed using VAS (visual analog scale) and/or Likert scale [134]. Pain, in association with stiffness was primarily assessed using WOMAC (Western Ontario and McMaster Universities osteoarthritis index) [135], but also KOOS (knee injury and osteoarthritis outcome score) [136], and/or ROM (range of motion) questionnaire [137]. As one of the first of such studies, Trock et al. additionally used a modified Ritchie scale to determine joint tenderness [138]. This or other joint indices represent a good measure to assess joint function and disease progression [139], and thus should be considered for further studies. Limitations in daily life were determined using the EQ-5D (EuroQol-5 Dimension) or OSW (Oswestry disability index) questionnaire [132,140]. Sutbeyaz et al. additionally used the NPDS (Northwick Park dependency score) [141] to determine the need for help in daily life [142].

Noteworthy, all studies have in common that ELF-PEMF treatment never negatively affected the reported outcome. However, the reported outcomes differ strongly, ranging from no effect to significant improvement by ELF-PEMF treatment. These great differences can neither be attributed to the patients’ age, gender, or BMI, nor the applied ELF-PEMF or treatment duration. However, ELF-PEMF effects seemed to be more pronounced when initial pain and disability levels were high and patients had demonstrable psychological strain. Thus, a possible explanation may be the mainly subjective nature of the primary outcome parameters. Therefore, it is recommended to additionally include more quantifiable measures in future studies.

One attempt was done by Reilingh et al., who investigated effects of ELF-PEMF treatment, applied for 4 hours per day over 60 days (∆t_b_ = n.s.; f_b_ = 75 Hz; peak amplitude intensity = 1.5 mT; ∆t_p_ = n.s.; generated with the I-ONE, IGEA, Carpi, Italy), in patients following arthroscopic debridement and micro-fracture of an osteochondral defect of the talus. In addition to the EQ-5D and the AOFAS (American Orthopaedic Foot and Ankle Society) score, the rate of sport resumption as well as the mean time to sport resumption were assessed. Most likely due to the relatively young mean age (~34 years) and the high activity level of the patients, this study could only detect a trend towards faster resumption to sport in the ELF-PEMF group [143].

This is in line with the study of Cadossi et al., who investigated the effect of ELF-PEMF treatment, applied for 4 hours per day over 60 days (∆t_b_ = n.s.; f_b_ = 75 Hz; peak amplitude intensity = 1.5 mT; ∆t_p_ = n.s.; generated with the I-ONE, IGEA, Carpi, Italy), in patients with osteochondral defects of the talus who received bone marrow-derived cell transplantation in the defect area. In this study, ELF-PEMF treatment did not affect the immediate outcome 30 days after surgery. However, patients in the ELF-PEMF group scored significantly better at the VAS and AOFAS scores than patients in the placebo group at the later follow-up time-points (2, 6, and 12 months) [144].

## 4. Conclusions

Our review clearly shows that ELF-PEMFs represent a valuable adjunct to conventional therapy for bone and osteochondral defects. However, treatment modalities have to be better defined in order to establish ELF-PEMF treatment in the clinical routine. Adjustment of the ELF-PEMF treatment conditions to specific indications (patients at high risk for complications, e.g., elderly, diabetics, smokers, etc.) and even phases of bone healing, which includes ELF-PEMF parameters (e.g. frequencies, intensities, pulse, and burst pattern, etc.), the way of application, as well as the frequency and the duration of the application are feasible. However, our understanding of the underlying mechanisms is still poor, which contributed to the fact that ELF-PEMF treatment to support bone healing and function could not be established in the clinical routine yet [154]. To accomplish this aim, in vitro research investigating the underlying mechanisms requires more holistic/comprehensive cell model approaches (e.g., three dimensional (3D) co-culture models) with sound parameter control resembling the in vivo situation in diseased or fractured bone. Only if the underlying mechanisms are better understood and this knowledge can be translated into the clinic, an indication-oriented treatment regimen using ELF-PEMFs will become a feasible therapeutic option. Therefore, more systematic studies are absolutely essential to unravel optimal treatment conditions.

## 5. Search Strategy

On the 18th of June 2019, a search was performed with PubMed and Web of Science. The search strategy is summarized in Table 2.

**Figure 8:**
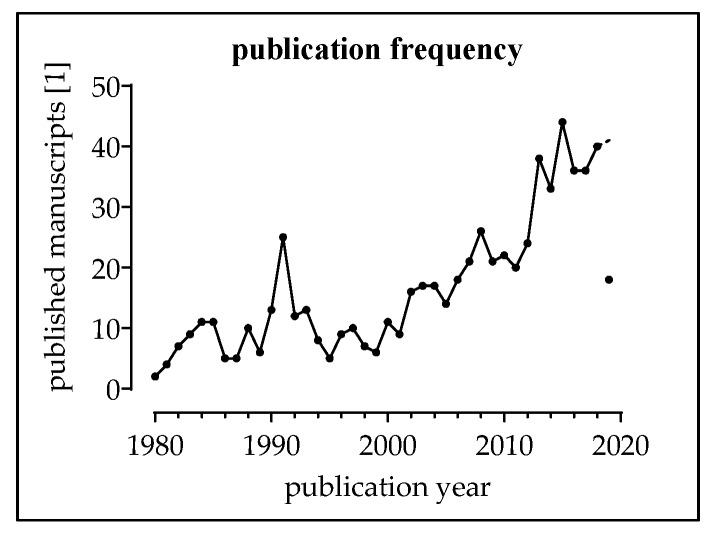
Number of publications published per year with the used (Table 2) search terms.

Considering only manuscripts in English or German language, a total of 662 manuscripts remained for further screening. In Figure 8, the number of papers published per year is presented.

## Figures and Tables

**Table 1:** Clinical studies (prospective, placebo-controlled, blinded) investigating the effect of ELF-PEMF treatment on osteoarthritis of the knee (adjunct to conservative treatment).

#	Year	Ref	Frequency	Peak Amplitude Intensity	Pulse & Burst Pattern	Daily PEMF Exposure	Treatment Period	all	+	−	Mean Age	Follow-Up	Compared to Placebo			
			f										Pain	Stiffness	Mobility	Quality of Life
1	1993	[145]	5–12 Hz	n.s.	n.s.	4.5 * 30 min/week	4 weeks	25	13	12	≥ 60 years	2, 4, 8 weeks	VAS ↘ motion ↘	n.s.	n.s.	n.s.
2	1994	[138]	5–12 Hz	n.s.	n.s.	4.5 * 30 min/week	4 weeks	86	42	44	67 years	2, 4, 8 weeks	VAS ↘ motion ↘	n.s.	n.s.	n.s.
4	2001	[146]	3, 7.8, 20 Hz ^1^	< 50 µT	n.s.	3 * 10 min/d	6 weeks	69	34	35	63 years	2, 4, 6 weeks	WOMAC ↘	WOMAC ↘	WOMAC ↘	EQ-5D ↗
5	2005	[147]	50 Hz ^2^	n.s.	∆t_p_ = 6 ms	5 * 2 h/week	6 weeks	83	42	41	60 years	2, 12 weeks	WOMAC =	WOMAC ↘	WOMAC =	n.s.
6	2005	[148]	10–300 Hz	13.6 µT	n.s.	16 min/d	6 weeks	71	35	36	60.2 years	6 weeks	KSS ↘	KSS ↘	KSS ↘	n.s.
7	2006	[149]	5 Hz/10 Hz ^3^	1.3–2.1 T	∆t_p_ = 270 µs	3 * 15 min/week	3 weeks	36	17	19	74,5 years	3, 4, 7 weeks	NRS ↘	OSW ↘	OSW ↘	n.s.
8	2006	[142]	0.1–64 Hz ^4^	n.s.	n.s.	2 * 30 min/d	3 weeks	32	17	15	42.5 years	3 weeks	VAS ↘	ROM ↗	mobility ↗	NPDS ↘
9	2009	[150]	50 Hz ^4^	105 µT	n.s.	5 * 30 min/week	3 weeks	55	30	25	58 years	3 weeks	VAS = Likert =	Lequesne = ROM =	Lequesne =	n.s.
10	2010	[151]	50 Hz ^5^	3 mT	T_b_ = 90 s	5 * 30 min/week	2 weeks	40	20	20	61.3 years	2 weeks	VAS = WOMAC =	WOMAC =	WOMAC =	n.s.
11	2013	[152]	6–100 Hz ^6^ (0.1–3 kHz)	n.s.	n.s.	3 * 20 min/week	6 weeks	28^#^	28	28	69.9 years	3 months	VAS ↘ WOMAC ↘	WOMAC ↘ ROM ↗	WOMAC ↘	n.s.
12	2016	[153]	50 Hz ^7^	100 µT	n.s.	5 * 60 min/week	4 weeks	29	14	15	57.2 years	4 weeks	VAS = WOMAC =	WOMAC =	WOMAC =	n.s.

^1^ Medicur, Snowden Healthcare, Nottingham, UK; ^2^ pulse generator, Biofields Aps, Copenhagen, Denmark; ^3^ CR-3000 system, CR Technology Co., Kyungki-do, Korea; ^4^ wave ranger professional, MRS 2000+Home, Eschen, Germany; ^5^ Energy Plus Roland Serie, Elettronica Pagani, Milan, Italy; ^6^ Magnetofield device, F&B International, Parma, Italy; ^7^ Automatic PMT Quattro Pro, ASA magnetic field, Arcugnano, Italy; ^#^ left–right comparison; + ELF-PEMF treatment; − placebo treatment; n.s.: not specified; $\Delta t_{p}$: pulse width; T_b_: burst repetition period; ↗ increased; ↘ decreased; = comparable; ROM: range of motion; VAS: visual analogue scale; WOMAC: Western Ontario and McMaster Universities osteoarthritis index; EQ-5D: EuroQol-5 Dimension; OSW: Oswestry disability index; NPDS: Northwick Park dependency score.

**Table 2:** Search strategy.

	Search Terms	All	Reviews	Clinical
**1**	“PEMF” or “pulsed electromagnetic” and “bone”	554	70	45
**2**	“PEMF” or “pulsed electromagnetic” and “osteopenia”	56	9	11
**3**	“PEMF” or “pulsed electromagnetic” and “osteoporosis”	85	12	11
**4**	“PEMF” or “pulsed electromagnetic” and “osteomalacia”	0	0	0
**5**	“PEMF” or “pulsed electromagnetic” and “fracture”	230	40	22
**6**	“PEMF” or “pulsed electromagnetic” and “non-union”	14	1	2
**7**	“PEMF” or “pulsed electromagnetic” and “pseudarthrosis”	29	5	4
**8**	“PEMF” or “pulsed electromagnetic” and “osteolysis”	5	0	0
**9**	“PEMF” or “pulsed electromagnetic” and “osteoarthritis”	83	22	22
**10**	“PEMF” or “pulsed electromagnetic” and “osteogenesis”	124	10	4
**11**	“PEMF” or “pulsed electromagnetic” and “osteogenic”	67	2	1
**12**	“PEMF” or “pulsed electromagnetic” and “MSC”	17	3	0
**13**	“PEMF” or “pulsed electromagnetic” and ”mesenchymal * cells”	70	3	1
**14**	“PEMF” or “pulsed electromagnetic” and “osteoblast”	124	6	0
**15**	“PEMF” or “pulsed electromagnetic” and “osteocyte”	9	1	0
**16**	“PEMF” or “pulsed electromagnetic” and “osteoclastogenesis”	130	11	4
**17**	“PEMF” or “pulsed electromagnetic” and “osteoclast”	34	2	0
**Sum**		1631	197	127
**Removal of Duplicates**		692	93	68
**plus Manuscripts from other sources**		710	97	81

PEMF: pulsed electromagnetic fields. MSC: mesenchymal stem cells.

## Abbreviations

ALP	alkaline phosphatase
BMD	bone mineral density
BMP	bone morphogenetic protein
COL	collagen
ECM	extracellular matrix
ELF	extremely low frequency
ELF-PEMF	extremely low frequency pulsed electromagnetic field
ELF-REMF	extremely low frequency rectangular electromagnetic field
ELF-SEMF	extremely low frequency sinusoidal electromagnetic field
ELF-TEMF	extremely low frequency triangular electromagnetic field
FN	fibronectin
KOOS	knee injury and osteoarthritis outcome score
MMP	matrix metalloproteinase
NPDS	Northwick Park dependency score
OP	surgery
OR	Odds Ratio
OSW	Oswestry disability index
ROM	range of motion
ROS	reactive oxygen species
TGF-β	transforming growth factor beta
UV	ultraviolet
VAS	visual analogue scale
VLF	very low frequency
WOMAC	Western Ontario and McMaster Universities osteoarthritis index
